# Notes and News

**Published:** 1891-08-08

**Authors:** 


### NOTES AND NEWS.

COLLECTED AND COMMUNICATED.

The Convalescent Home at Seaford was formally opened on the 27th ult. by the Duke of Cambridge. It was decided that the ceremony should be a private one, and no popular demonstration took place; nevertheless, a large concourse, consisting of the Committee, the trustees, and their friends, were present. The Home, which will supply a most important want, is open to the whole of the county of Surrey. The Duke of Cambridge is President, and the Duke of Connaught is Vice-President of the Home.

The sum of £20,000 for a new hospital is an expenditure for a county like Fife to undertake with no light heart. The Lunacy Board were practically forced into the course they have adopted. It is an undertaking that has been laid upon the asylum authorities by the stern necessities of an increasing asylum population. The proposal has been discussed and deliberated on with the utmost care for fourteen months, with the result that the Building Committee are absolutely unable to bring down the total outlay to less than £20,000.

A meeting of the Manchester Royal Infirmary Board was held at the Infirmary on the 27th ult., Mr. E. S. Heywood presiding. The minutes of the House Committee were read, and included the report of the Chloroform Fatalities Subcommittee. The Committee stated: "We have to report —1. That of the five deaths referred to by the Coroner at the recent inquest, two only were due to the direct effects of the anæsthetic, and that, of the other three, two were due to vomited matter getting into the larynx and producing suffocation, and one to collapse suddenly supervening after the operation had been concluded and the patient had nearly recovered from the effects of the anæsthetic. 2. We have been informed by Mr. Alexander Wilson, the anæsthetist, that he has used the chloroform in question about 800 times, and had observed nothing unusual in the behaviour of the patients either during or after its administration. 3. We have learned that that chloroform is largely used in other hospitals, and that no difference has been observed between it and others. 4. A report on the quality of the chloroform, by Professor Attfield, was laid before us, in which it is stated that 'it possesses all the character and responds to all the tests of pure chloroform of the British Pharmacopœia, and, in short, is a perfectly pure chloroform.' He also certifies that it is 'of excellent quality for anæsthetic purposes.' 5. Under these circumstances, we are satisfied that the chloroform in question is a good and reliable article, that the deaths were not attributable to any peculiarity belonging to it, but would probably have equally occurred under the use of chloroform manufactured by any other process; and we further see no reason why the use of this chloroform should not be continued in the hospital."

The British Medical Association held its fifty-ninth annual meeting at Bournemouth, last week, under the presidency of Dr. J. Roberts Thomson. The meeting was highly successful, the address of Dr. Lauder Brunton on medicine being specially noteworthy. It was determined, despite the strenuous opposition of Dr. Drage and others, to instruct the Parliamentary Committee to accept and support any measure introduced into Parliament for the registration of midwives by the State, which received the approval of the majority of the branches of the association.

If the adage that there is no smoke without fire be true, it will be proved that ground for the numerous complaints against the Preston Workhouse exists, after all. It appears that the Local Government Board have received a petition, with eighty signatures appended, asking for an enquiry into the working of the Union Workhouse at Fulwood. The Local Government referred to the Board of Guardians, upon which a meeting was called. The Board decided to reply to the Local Government Board to the effect that they courted an enquiry into the complaints made against the management of the workhouse. In the course of discussion, it was put forward that the dissatisfaction emanated from some of the Guardians themselves, who were unfavourably disposed towards Mr. and Mrs. Bateman, Master and Matron of the Workhouse.

The strides which hygienic science has made of late years, and the interest which is taken by the public in it, has no more striking proof than the continued holding of the International Congress of Hygiene and Demography, which for the last seven years has met in various Continental cities. This year the English metropolis is to be the scene of this gathering, and we have no doubt that for the interest of its proceedings and the attendance of members it will show no falling off when compared with its predecessors. The President of the Congress is the Prince of Wales, who is to preside at the inaugural meeting on the afternoon of the 10th inst. at St. James's Hall. During the four following days papers will be read on a variety of subjects at Burlington House, the University of London, and at the Royal School of Mines, Jermyn Street, the subjects including preventive medicine, bacteriology, the relation of the diseases of animals to those of man, the hygiene of infancy and childhood, chemistry and physics, architecture, and engineering in relation to hygiene, naval and military and State hygiene, demography, health statistics, and industrial hygiene. Among the papers to be read which relate more directly to hospitals are those on the improved hygienic conditions of maternity hospitals, (by Dr. Priestley); the hospital ambulance organisation of the Metropolitan Asylums Board for the removal and isolation of infectious diseases (by Deputy Surgeon Bostock, C.B., and Sir Vincent Kennett Barrington); the Architecture of English Isolation Hospitals (by Dr. Thorne Thorne); the Heathcote Hospital, Leamington (by Mr. Keith D. Young); Hospital Tents and Huts (Dr. Duchausoy); Temporary Local Isolation Hospitals as opposed to a Central Hospital (by the Rev. C. E. Few, Vicar of Seal, Sevenoaks). The time of the Congress will not, however, be wholly given up to the discussion of these matters, but a part will be spent in social functions, amongst these being conversaziones to be held by the Colleges of Surgeons and Physicians, an entertainment at the Guildhall by the Court of Common Council, and a garden party at Holly Lodge, given by Mr. and the Baroness Burdett-Coutts. Saturday, the 15th, will be devoted to excursions. The ladies, too, who accompany their husbands to London for the Congress will be received every day in Agnew's Gallery, Old Bond Street, by the Ladies' Committee; and while "their husbands are engaged in the more serious work of the sections" (we are quoting from the official programme), these ladies will be able to amuse themselves, as sight-seeing parties are to be organised daily. The closing meeting of the Congress is fixed for the 17th, in the theatre of the University of London. Members and ladies' tickets, and all further information, may be obtained from the Secretary, 20, Hanover Square, W.C.

